# The Src inhibitor dasatinib accelerates the differentiation of human bone marrow-derived mesenchymal stromal cells into osteoblasts

**DOI:** 10.1186/1471-2407-10-298

**Published:** 2010-06-17

**Authors:** Hichame Id Boufker, Laurence Lagneaux, Mehdi Najar, Martine Piccart, Ghanem Ghanem, Jean-Jacques Body, Fabrice Journé

**Affiliations:** 1 Laboratoire d'Hématologie Expérimentale, Institut Jules Bordet, Université Libre de Bruxelles, Brussels, Belgium; 2 Laboratoire d'Oncologie et de Chirurgie Expérimentale, Institut Jules Bordet, Université Libre de Bruxelles, Brussels, Belgium; 3 Clinique d'Oncologie Médicale, Institut Jules Bordet, Université Libre de Bruxelles, Brussels, Belgium; 4 Service de Médecine, CHU Brugmann, Université Libre de Bruxelles, Brussels, Belgium

## Abstract

**Background:**

The proto-oncogene Src is an important non-receptor protein tyrosine kinase involved in signaling pathways that control cell adhesion, growth, migration and differentiation. It negatively regulates osteoblast activity, and, as such, its inhibition is a potential means to prevent bone loss. Dasatinib is a new dual Src/Bcr-Abl tyrosine kinase inhibitor initially developed for the treatment of chronic myeloid leukemia. It has also shown promising results in preclinical studies in various solid tumors. However, its effects on the differentiation of human osteoblasts have never been examined.

**Methods:**

We evaluated the effects of dasatinib on bone marrow-derived mesenchymal stromal cells (MSC) differentiation into osteoblasts, in the presence or absence of a mixture of dexamethasone, ascorbic acid and β-glycerophosphate (DAG) for up to 21 days. The differentiation kinetics was assessed by evaluating mineralization of the extracellular matrix, alkaline phosphatase (ALP) activity, and expression of osteoblastic markers (receptor activator of nuclear factor kappa B ligand [RANKL], bone sialoprotein [BSP], osteopontin [OPN]).

**Results:**

Dasatinib significantly increased the activity of ALP and the level of calcium deposition in MSC cultured with DAG after, respectively, 7 and 14 days; it upregulated the expression of BSP and OPN genes independently of DAG; and it markedly downregulated the expression of RANKL gene and protein (decrease in RANKL/OPG ratio), the key factor that stimulates osteoclast differentiation and activity.

**Conclusions:**

Our results suggest a dual role for dasatinib in both (i) stimulating osteoblast differentiation leading to a direct increase in bone formation, and (ii) downregulating RANKL synthesis by osteoblasts leading to an indirect inhibition of osteoclastogenesis. Thus, dasatinib is a potentially interesting candidate drug for the treatment of osteolysis through its dual effect on bone metabolism.

## Background

Osteoblasts originate from mesenchymal osteoprogenitor cells and play a key role in physiological bone turnover and pathological disorders including osteoporosis [1], Paget's disease [2] and tumor-induced osteolysis [3]. Osteoblast functions are dependent on their differentiation status. Indeed, immature osteoblasts regulate recruitment, differentiation and maturation of osteoclasts [4], as well as activity of osteoclasts [5]. By contrast, mature osteoblasts produce bone matrix (collagen synthesis and mineralization) [6]. Thus, the control of osteoblast differentiation is critical in the management of bone diseases.

In recent years, much interest emerged for the bone marrow-derived mesenchymal stromal cells (MSC) due to their ability to self-renew, proliferate and differentiate into a variety of cell types of mesodermal, endodermal and ectodermal origins [7]. There are no specific markers of MSC but these cells can be selected on the basis of a complex immunophenotype, comprising the differential expression of cell surface molecules (CD29, CD73, CD90, CD105 and CD166), and of markers of hematopoietic stem cells (CD34, CD45) and endothelial cells (CD31) [8]. MSC exhibit various phenotypic characteristics of osteoblasts and can be grown in culture to differentiate into mature osteoblasts able to form mineralized bone nodules [9,10]. Recent studies have demonstrated successful osteogenic differentiation of MSC following treatment with bone morphogenetic proteins (BMP)-2,-4,-6 [11], parathyroid hormone (PTH) plus vitamin D3 [12], transforming growth factor beta 1 (TGFβ1) [13], estrogens [14], and also oxysterols [15]. On the other hand, the combination of dexamethasone, ascorbic acid and β-glycerophosphate (DAG) remains the most widely used tool to induce differentiation of MSC into osteoblasts [16], but specific markers of the osteoblast lineage, especially during the early stages of differentiation, remain to be uncovered.

The proto-oncogene Src is a member of the Src family kinases (SFK) and has important roles in physiological and pathological processes such as cell survival, differentiation, tumorigenesis and inflammation [17]. Src kinase is regulated by growth factors, cytokines, cell adhesion, and antigen receptor activation [18]. It is generally maintained in an inactive conformation by phosphorylation at ^527^Tyr. The dephosphorylation of this residue by phosphatases leads to intramolecular autophosphorylation at ^416^Tyr, promoting the kinase activity [19]. Src signaling coordinates both osteoclast and osteoblast activities [20]. Recent studies have reported that Src kinase plays a positive role in osteoclast survival and resorbing activity, including cytoplasm polarization and ruffled border formation [21]. On the other hand, Src may negatively regulate osteoblast maturation through a mechanism where the cytoplasmic shuttling Yes-associated protein (YAP) is recruited on the runt-related transcription factor 2 (Runx2) nuclear domains to inhibit expression of Runx2 regulated genes [22]. Thus, Src kinase is essential for osteoclast activation and osteoblast inhibition [20,23], and stands out as a promising therapeutic target for the prevention and the treatment of bone loss.

Dasatinib (BMS-354825) is a new dual Src/Bcr-Abl tyrosine kinase inhibitor. It was originally developed for the treatment of patients with chronic myeloid leukemia (CML) associated with a reciprocal translocation between chromosomes 9 and 22 that results in the formation of the Philadelphia chromosome and constitutively active tyrosine kinase Bcr-Abl [24]. It has recently been used for the treatment of imatinib-resistant CML [25]. Besides CML, dasatinib, by acting as a Src kinase inhibitor, has shown promising results in preclinical studies in various solid tumors. A recent study using non-small cell lung cancer and head and neck squamous cell carcinoma cells has shown that it can inhibit cell migration and invasion, arrest cell cycle, and induce apoptosis [26]. In prostate cancer cells, dasatinib was reported to block the kinase activity of Src and inhibit tumor cells adhesion, migration and invasion [27]. It is also able to decrease tumor size and reduce the metastatic potential of pancreatic cancer cells in an orthotopic nude mouse model [28]. In addition, very recent data revealed that dasatinib strongly promoted differentiation of primary mouse osteoblasts isolated from mouse calvaria [29].

In the present study, we evaluated the effects of the Src inhibitor dasatinib on osteogenesis using an in vitro model of osteoblast differentiation comprising human bone marrow-derived MSC treated or not with DAG. To this aim, we first identified each stage of the differentiation using a panel of specific markers, and then, we examined the effects of dasatinib on these markers.

## Methods

### Selection and culture of human bone marrow-derived mesenchymal stromal cells

Bone marrow was harvested from the sternum of healthy volunteer donors (n = 7) and from the iliac crest of donors of bone marrow for transplantion (n = 3). The mean age of the donors was 25 years (range 5-55). Informed consent was obtained from all donors. The ethics committee of our Institution approved the use of the tissue material for this study.

Mononuclear cells (MNC) were isolated by density gradient centrifugation (Linfosep, Biomedics, Madrid, Spain) and washed in HBSS medium (BioWhittaker, Walkersville, MD). MNC were seeded at 5 × 10^6 ^cells/cm^2 ^in alpha-minimum essential medium (α-MEM, BioWhittaker) supplemented with 15% FBS, 2 mM L-glutamine, 0.5% antibiotic/antimycotic solution (all from Gibco-BRL, Life Technologies, Merelbeke, Belgium). Cells were incubated at 37°C in a humidified 95% air and 5% CO_2 _atmosphere, cultured up to 80% confluence, and then trypsinized (trypsin-EDTA solution, Gibco-BRL), centrifuged, and replated at a density of 200 cells/cm^2 ^for all subsequent passages. All experiments were performed with cells after the second or the third passage.

### Colony Forming Unit (CFU-F) assay

CFU-F assay was used to evaluate the number of mesenchymal progenitors in fresh bone marrow and after each passage. 10^5 ^MNC or 5,000 cells obtained after each passage were incubated in Petri dishes in a complete α-MEM medium. Ten days later, fibroblastic colonies with more than 50 cells were counted under light microscopy after a May Grunwald Giemsa coloration (Merck, Darmstadt, Germany).

### Flow cytometry analysis

MSC phenotype was evaluated after each passage by the expression of CD31 (Immunotech, Marseille, France), CD34 (BD Biosciences Pharmingen, San Diego, CA), CD29 (Immunotech), CD45 and HLA-DR (Exalpha Biologicals, Maynard, MA), CD166 (DakoCytomation Denmark A/S, Glostrup, Denmark), CD73 (BD Biosciences Pharmingen) and CD105 (RD Systems, Minneapolis, MN). Cells were incubated for 30 min at room temperature with primary PE- (phycoerythrine) or FITC- (fluoresceine isothiocyanate) conjugated antibodies. Flow cytometry was performed using a Coulter EPICS XL (Beckman-Coulter, Miami, FL); 5,000 events for each sample were recorded.

### Induction of osteogenic differentiation

MSC were seeded at 2,500 cells/cm^2 ^in 24-well dishes (for calcium & ALP assays) or in Petri culture plates (for RT-PCR) and cultured in α-MEM supplemented with 15% FBS up to confluence. Osteoblastic differentiation of MSC was induced by incubation with 10^-7 ^M dexamethasone (Aacidexam^®^, Aaciphar, Brussels, Belgium), 6 × 10^-5 ^M ascorbic acid (Sigma, St Louis, MO) and 10^-2 ^M β-glycerophosphate (Sigma) (=DAG) [16] for up to 3 weeks. Dasatinib, provided by Bristol Myers Squibb (Princeton, NJ), was solubilized in dimethyl sulfoxide (DMSO) (stock solution 10^-1 ^M) and used at 10^-8 ^M alone or in combination with DAG (as specified in Results). E804 (indirubin-3'-(2,3-dihydroxypropyl)oximether, Alexis Biochemicals, Enzo Life Sciences BVBA, Zandhoven, Belgium), a specific Src inhibitor, was solubilized in DMSO (stock solution 10^-2 ^M) and used at 10^-7 ^M alone or in combination with DAG. The osteogenic medium was changed weekly and experiments were stopped after 7, 14 or 21 days to assess the osteoblastic phenotype of MSC. Osteogenic differentiation was monitored by mineralization assay, alkaline phosphatase activity measurement, osteoblast-related gene expression determination, and RANKL/OPN ratio ELISA evaluation.

### Quantitative determination of calcium accumulation

To evaluate calcium deposition, the matrix was demineralized by addition of 500 μl of 0.6 N HCl during an overnight incubation at 37°C. Solutions were then collected and centrifuged at 2,000 × g for 5 min. Calcium concentration in the supernatant was determined by colorimetry (QuantiChrom Calcium Assays Kit, BioAssay Systems, Hayward, CA) as described by the manufacturer. Briefly, 5 μl samples were combined with 200 μl calcium reagent and incubated for 5 min at room temperature. The absorbance was measured immediately after incubation at 610 nm using a plate reader (Organon Teknika Cappel Products, West Chester, PA).

### Quantitative analysis of alkaline phosphatase activity

ALP activity was determined using the LabAssay™ ALP (Wako Chemicals Gmbh, Neus, Germany), according to the manufacturer's recommendations. This measurement consists of the determination of the quantity of p-nitrophenol released from the substrate. The cell layers were lysed with 100 μl of ice-cold 0.1% Triton X-100 in PBS. Samples were then frozen and thawed twice, and the cell lysates were collected. Samples (20 μl) were combined with 100 μl ALP reagent and the activity was measured after an incubation of 15 min at 37°C. The absorbance was measured immediately at 405 nm and the amount of p-nitrophenol was determined by comparison with a standard curve. ALP activity (U, μmol p-nitrophenol released per min) was normalized to the number of cells evaluated by the trypan blue dye exclusion assay.

### Semi-quantitative RT-PCR assay

Total RNA from MSC cultured in Petri plates was extracted by the Tripure method (Roche Diagnostics, Indianapolis, IN). The samples (1 μg RNA) were treated by DNAse (Invitrogen Life Technologies, Merelbeek, Belgium) at 37°C for 30 min in a final volume of 10 μl containing 1 μl DNAse, 10 × buffer and 1 U DNAse RQI. The reverse transcription was performed with 1 μg DNAse-treated RNA using the M-MLV reverse transcriptase (Invitrogen Life Technologies) in a final volume of 20 μl containing 4 μl first-strand buffer, 10^-2 ^M DTT, 1 μM each of dNTP, 50 URNase inhibitor, and 100 U M-MLV reverse transcriptase, leading to the production of cDNA. Five ng cDNA were used for PCR reaction in a final volume of 50 μl containing 25 μl multiplex PCR mix and 200 nmol forward and reverse primers (Sigma-Genosys, Pampisford Cambs, U.K.) (see list in Table 1). After the activation step (95°C for 15 min), each cycle consisted of denaturation at 94°C for 30 sec, annealing at 60°C for 90 sec, extension at 72°C for 90 sec and elongation at 72°C for 10 min. After the optimal number of cycles determined for each primer set, PCR products were separated by electrophoresis on 2% (w/v) agarose gel and were visualized by ethidium bromide staining. Gels were scanned with FLA-5000 imaging system (Fujifilm, Tokyo, Japan) and Image Reader software (Raytest^®^, Straubenhardt, Germany). Band intensities were quantified using AIDA^® ^Image Analyser 3.45 software (Raytest^®^) and normalized against β-actin mRNA used as an internal control.

**Table 1:** Primers used for semi-quantitative RT-PCR evaluation of RNA of osteoblast-related genes and housekeeping gene.

Transcript name		RT-PCR primer set (5'-3')	Product length
β-actin	Forward Reverse	tgacggggtcaccacactgtgcccatcta ctagaagcatttgcggtggacgatggaggg	610
ALP	Forward Reverse	cagaagctcaacaccaacg ccagcaagaagaagcctttg	815
BMP2	Forward Reverse	cccacttggaggagaaacaa acgtctgaacaatggcatga	351
BSP	Forward Reverse	atggcctgtgctttctcaat ccgtttatgccttgttcgtt	497
Runx2	Forward Reverse	aagaaggacagacagaagc aggtggcagtgtcatcatct	428
COL-I	Forward Reverse	agtgctagacatgctcagct ttcaccatctctgcctgcgg	173
OPG	Forward Reverse	tgcagtacgtcaagcaggag gtgtcttggtcgccattttt	754
OPN	Forward Reverse	ctaggcatcacctgtgccatacc cagtgaccagttcatcagattcatc	395
OSN	Forward Reverse	gtgcagaggaaaccgaagag tcattgctgcacaccttctc	172
OSX	Forward Reverse	ggcacaaagaagccgtactc gccttgtaccaggagccata	296
PTHr	Forward Reverse	aggaacagatcttcctgctgca tgcatgtggatgtagttgcgcgt	521
RANKL	Forward Reverse	agagcgcagatggatcctaa ttccttttgcacagctcctt	180

### Real-time quantitative PCR assay

Messenger RNA expressions of relevant osteoblast-related markers (RANKL, OPG, BSP, OPN) were quantified by RTqPCR using SYBR^® ^Green dye (SYBR^® ^Green PCR Master Mix, Applied Biosystems, Foster City, CA) and sequence-specific primers (Table 2). Total RNA from control and treated cells was isolated using Tripure method (Roche Diagnostics). Reverse transcription was performed using 1 μg total RNA and the reverse transcription system (Promega, Madison, WI). The amplification was performed in an ABI PRISM^® ^7900 Sequence Detection System (Applied Biosystems) with 40 cycles of a two-step PCR (95°C for 15 sec and 60°C for 60 sec) after an initial activation step (95°C for 10 min). Melting curves from 60°C to 99°C were assessed to evaluate specificity. Serial dilutions of purified amplicons served to generate standard melting curves. Relative quantification was calculated by normalizing the test crossing thresholds (Ct) with the β-actin amplified control Ct.

**Table 2:** Primers used for real-time quantitative RT-PCR evaluation of RNA of RANKL, OPG, BSP, OPN and β-actin.

Transcript name		RT-PCR primer set (5'-3')	Product length
β-actin	Forward Reverse	ctggcacccagcacaatg ccgatccacacggagtacttg	68
BSP	Forward Reverse	tacacgggcgtcaatgaata aggttccccgttctcacttt	63
OPG	Forward Reverse	ggcaacacagctcacaagaa cgctgttttcacagaggtca	117
OPN	Forward Reverse	ttgcagtgatttgcttttgc gccacagcatctgggtattt	115
RANKL	Forward Reverse	agagcgcagatggatcctaa ttccttttgcacagctcctt	180

### Cell proliferation assay

Cell proliferation was assessed by a colorimetric assay based on the cleavage of the tetrazolium salt WST-1 to a formazan-class dye by the mitochondrial succinate-tetrazolium reductase in viable cells. Briefly, MSC were seeded in 96-well plates (density 1,000 cells/well) in complete culture medium and cultured for 24 hours. Cells were then exposed to increasing concentrations of dasatinib or vehicle, in presence or not of DAG, for 3, 7 or 10 days as described in "Results". Then, the Cell Proliferation Reagent WST-1 (Roche Molecular Biochemicals, Mannheim, Germany) was added to the plate, and the cells were cultured for 2 additional hours. The quantity of formazan dye, directly related to the number of metabolically active cells, was quantified by measuring the absorbance at 450 nm with a multiwell spectrophotometer (Organon Teknica, Austria). Blank wells lacked cells and drugs.

### Western blotting

Control and dasatinib-treated MSC (cultured in Petri plates) were lysed using detergent cocktail (M-PER Mammalian Extraction Buffer) supplemented with protease inhibitors (Halt Protease Inhibitor Cocktail) and phosphatase inhibitors (Halt Phosphatase Inhibitor Cocktail) (all from Pierce, Rockford, IL, USA). Protein concentrations were determined by the BCA Protein Assay (Pierce) using bovine serum albumin as standard. Equal amounts of cell proteins (30 μg) were subjected to 10% SDS-PAGE and electrotransferred onto nitrocellulose membranes (Amersham Pharmacia Biotech, Roosendaal, The Netherlands). Immunodetections were performed using a rabbit polyclonal anti-human p-Src (Tyr 419) antibody (1:200) (Santa Cruz Biotechnology, Santa Cruz, CA, USA) or a rabbit polyclonal anti-human Src antibody (1:1,000) (Cell Signaling Technology, Danvers, MA, USA). Peroxidase-labeled anti-rabbit IgG antibody (1:5,000) (from Amersham Pharmacia Biotech) was used as secondary reagents. Bound peroxidase activity was revealed using the SuperSignal^® ^West Pico Chemiluminescent Substrate (Pierce). Immunostaining signals were digitalized with a PC-driven LAS-3000 CCD camera (Fujifilm, Tokyo, Japan), using a software specifically designed for image acquisition (Image Reader, Raytest^®^, Straubenhardt, Germany).

### Enzyme-linked immunosorbent assay (ELISA)

Measurements of RANKL and OPG in 24-hours conditioned medium of MSC (cultured in Petri plates) exposed to dasatinib for 3 and 7 days were performed using two commercially available ELISA kits (ampli-sRANKL ELISA and Osteoprotegerin ELISA, Biomedica GmbH, Vienna, Austria) according to the manufacturer's instructions. Briefly, 50 μl conditioned medium (1 ml corresponding to 10^5 ^cells) or Standards, 50 μl polyclonal biotinylated antibodies, and 100 μl Assay Buffer were incubated for 24 hours at 4°C in microtiter plates pre-coated with human recombinant OPG (for RANKL detection) or monoclonal anti-OPG antibody (for OPG detection). The wells were washed with 300 μl Wash Buffer and were incubated with 200 μl Conjugate for 1 hour at room temperature. Then, the wells were incubated with Substrate for 20 min at room temperature and the absorbance was measured using a plate reader (Organon Teknika Cappel Products) at 450 nm with reference at 620 nm after the addition of 50 μl Stop Solution. Standard ranges were 0-2 pmol/L for RANKL and 0-30 pmol/L for OPG. Detection limits were 0.02 pmol/L for RANKL and 0.14 pmol/L for OPG. Data were normalized to total protein content (BCA Protein Assay, Pierce) and were presented as RANKL/OPG ratio.

### Statistical analysis

Results are expressed as the mean ± the standard error of the mean (SEM) of independent experiments (one experiment used bone marrow-derived MSC from one single donor), each performed in duplicate. Statistical analysis was performed by analysis of variance (ANOVA). Tukey post hoc test was used for multiple comparisons between groups. Statistical significance was set at * 0.05, ** 0.01 and *** 0.001. All analyses used SPSS software (Paris, France).

## Results

### Isolation and expansion of MSC

MSC cultures were initiated from 20 ml of bone marrow aspirates obtained from 10 healthy donors. After plating, cells showed monocyte-like and fibroblast-like morphology. During cell expansion, fibroblast-like cells (=MSC) became progressively predominant and proliferated up to confluence. To estimate the number of mesenchymal progenitors during expansion, the ability of MNC to form fibroblastic colonies were assessed using the colony forming unit - fibroblastic assay (CFU-F assay). The mean ± SEM number of CFU-F obtained from the 10 bone marrow samples was 22 ± 6 colonies/10^6 ^cells, confirming the low level of MSC in the bone marrow. After passage 1, the mean number of CFU-F significantly increased to 18 ± 4 × 10^4 ^colonies/10^6 ^cells. Importantly, CFU-F efficiency remained stable throughout the duration of the culture. In addition, the expression of MSC surface antigens was evaluated after each passage. Flow cytometry analysis revealed that the majority of adherent cells were positive for CD29, CD166, CD105 and CD73, and were negative for markers of the endothelial lineage CD31, of the hematopoietic lineage (CD34) and for the leukocyte common antigen CD45 and HLA-DR (=MHC-II molecule), confirming the selection of MSC.

### Evaluation of matrix mineralization, alkaline phosphatase activity and osteoblast-related gene expression in MSC exposed to DAG

**Figure 1:**
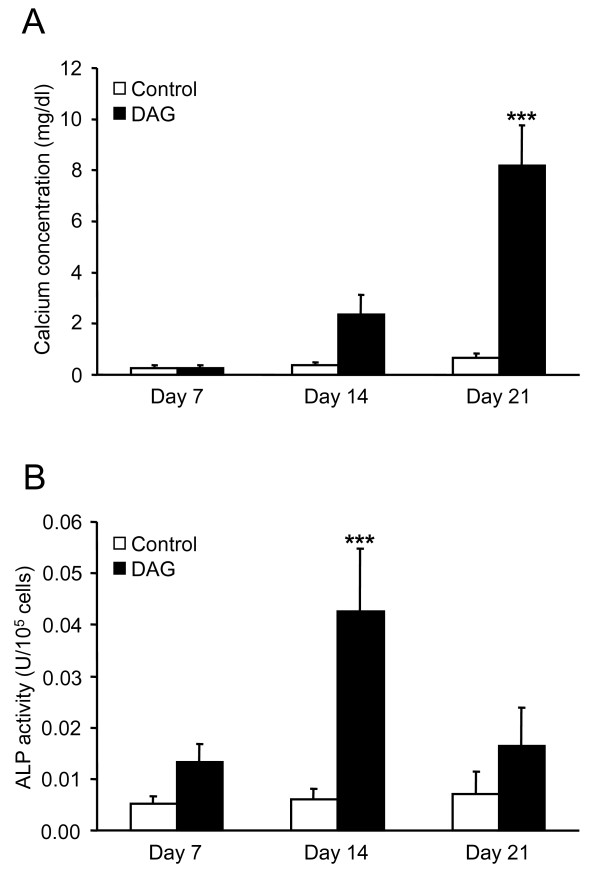
**Quantitative analysis of matrix calcification and alkaline phosphatase activity during osteogenic differentiation of MSC**. **A**: Calcium content of the extracellular matrix of control cell cultures (open bars) and DAG-treated cell cultures (black bars) was determined after 7, 14 and 21 days. **B**: ALP activity in MSC cultured in control (open bars) or osteogenic (black bars) medium was measured after 7, 14 and 21 days. ALP activity (U, μmol p-nitrophenol released per min) was normalized for 10^5 ^cells. **A**, **B**: Results are presented as mean ± SEM from 5 independent experiments performed in duplicate; *** p < 0.001 versus the corresponding control.

Calcium deposition was quantified by colorimetric assay (Fig. 1A). DAG treatment of MSC stimulated calcium deposition (increased by 6.5 and 12.3-fold compared to untreated MSC at days 14 and 21, respectively), confirming that DAG is able to induce differentiation of MSC into the osteogenic lineage up to matrix mineralization.

ALP activity was quantified with the LabAssay™ ALP (Wako Chemicals Gmbh, Neus, Germany). It was significantly increased in DAG-treated MSC: it increased at day 7 (by 2.6-fold versus untreated cells at day 0), reached a maximum at day 14 (7.1-fold) and decreased after 21 days (2.3-fold) of exposure to DAG (Fig. 1B). These data indicate that enhancement of ALP activity precede matrix mineralization.

**Figure 2:**
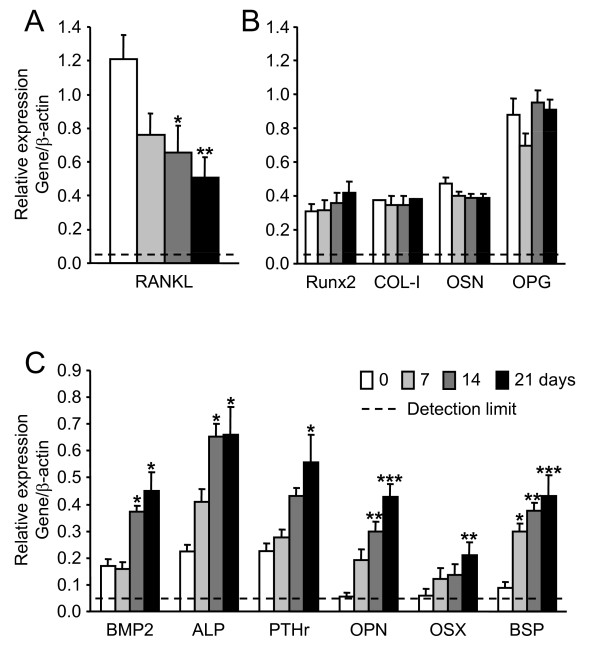
**Semi-quantitative evaluation of expression of osteoblast-related genes in MSC treated or not with DAG for 7, 14 and 21 days**. Gene expressions were divided into 3 groups. **A**: a gene whose expression was downregulated by DAG exposure, **B**: 4 genes whose expressions did not change under treatment, and **C**: 6 genes whose expressions were upregulated by treatment. **A**, **B**, **C**: Total RNA was isolated from MSC and subjected to semi-quantitative RT-PCR analysis using specific primers for osteoblast gene markers and β-actin (see Table 1). Amplified products were separated by electrophoresis, quantified by densitometry and results were corrected by comparison with the housekeeping gene. Data are presented as mean ± SEM from 5 independent experiments performed in duplicate; * p < 0.05, ** p < 0.01 and *** p < 0.001 versus the corresponding gene expression at time 0.

Changes in mRNA levels of osteoblast-related genes were examined using semi-quatitative RT-PCR during osteoblastic differentiation of MSC treated with DAG. We assessed gene expressions of (i) transcription factors known to be required for bone formation (Runx2, osterix [OSX]), (ii) hormones and receptors implicated in osteoblast activity (PTH receptor [PTHr], bone morphogenetic protein-2 [BMP-2], sclerostin [SOST]), (iii) extracellular matrix proteins and enzymes reflecting osteoblastic phenotype (type-I collagen [COL-I], bone sialoprotein [BSP], osteopontin [OPN], osteonectin [OSN], alkaline phosphatase [ALP]) and (iv) factors controlling osteogenesis (receptor activator of NFκ-B ligand [RANKL] and osteoprotegerin [OPG]). β-actin was used as housekeeping gene. The tested genes could be classified into three groups (Fig. 2). The first group included the RANKL gene whose expression was high in untreated cells and fell to lower levels during the whole process of osteoblast differentiation (Fig. 2A). The second group was composed of Runx2, COL-I, OSN and OPG genes whose expressions were already detectable in untreated MSC and were not significantly modified by DAG treatment (Fig. 2B). These gene markers cannot thus help to define the osteoblastic stage. The third group comprised BMP-2, ALP, PTHr, OPN, OSX and BSP genes whose expressions were not (BSP, OPN and OSX) or weakly detectable in untreated MSC, but significantly increased in DAG-treated cells to reach maximal values after 7 (BSP), 14 (BMP-2, ALP) or 21 days (PTHr, OPN, OSX) (Fig. 2C).

Of note, ALP activity (Fig. 1B) did not reveal comparable changes to the ALP gene (Fig. 2C) during the differentiation process of MSC induced by DAG. Indeed, no or low ALP activity was measured in undifferentiated MSC which expressed moderate levels of ALP gene, and ALP activity was significantly reduced in "late osteoblasts" which kept a relatively high expression level of ALP gene. Therefore, due to the specific variations in ALP activity during MSC differentiation into osteoblasts, we selected this parameter to characterize the differentiation steps.

**Figure 3:**
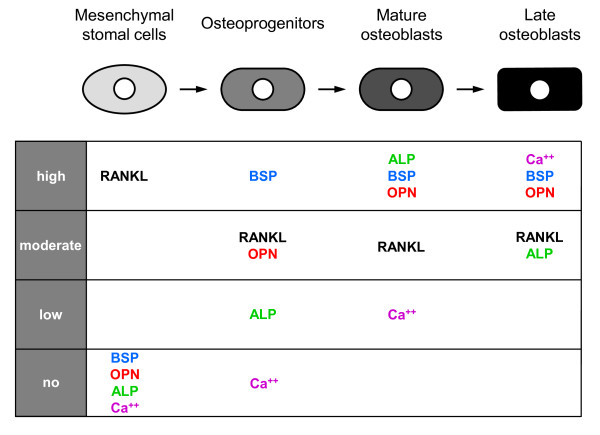
**Scheme indicating that a combination of 5 osteoblast-related parameter levels/activity (Ca^++ ^deposition, ALP activity, and RANKL, BSP, OPN gene expression) can be used as an indicator of the stage of MSC differentiation into osteoblasts**. Using this setting, 4 stages were defined: (i) undifferentiated MSC (=native stage) expressed high RANKL levels, but no BSP, no OPN, no ALP and no mineralization activity, (ii) osteoprogenitors (=early stage observed after 7 days of DAG treatment) expressed high BSP, moderate RANKL, moderate OPN levels, but had low ALP and no mineralization activity, (iii) mature osteoblasts (=intermediate stage reached after 14 days of DAG treatment) had a high ALP activity, expressed high BSP, high OPN, moderate RANKL levels and had low mineralization activity, and (iv) late osteoblasts (=late stage obtained after 21 days of DAG treatment) had a high mineralization activity, expressed high BSP, high OPN and moderate RANKL levels and had moderate ALP activity.

Thus, this part of the study allowed us to identify mineralization process, ALP activity and expression of RANKL, BSP and OPN as strong markers of osteoblastic differentiation of MSC since they were clearly changing between each stage of the differentiation process (Fig. 3). This model was further used to test the Src inhibitor dasatinib on MSC differentiation into osteoblasts.

### Effect of dasatinib on MSC proliferation

**Figure 4:**
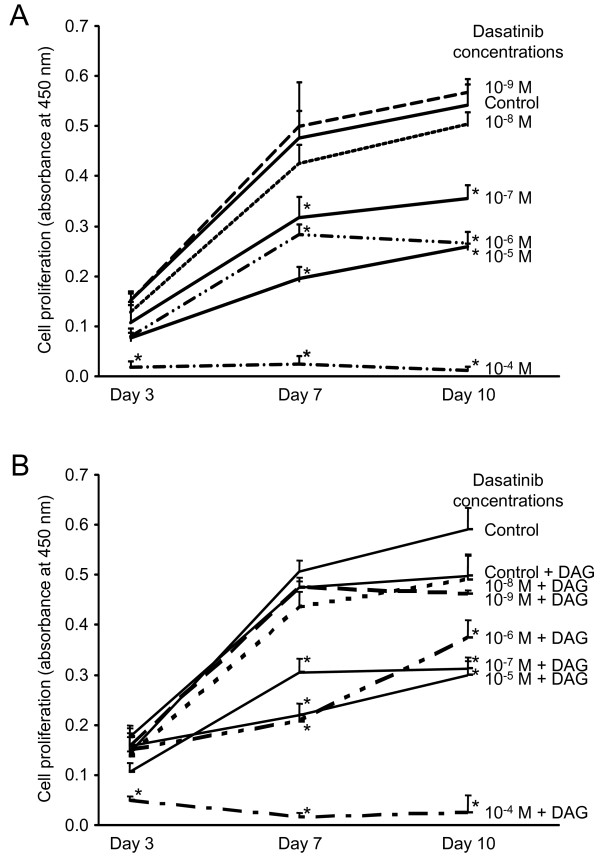
**Proliferation rate of MSC exposed to dasatinib alone or in combination with DAG**. **A**: Cells were treated for 3, 7 and 10 days with increasing concentrations of the drug (10^-9 ^- 10^-4 ^M) or vehicle (Control) in complete culture medium. **B**: Cells were exposed to increasing concentrations of the dasatinib (10^-9 ^- 10^-4 ^M) or vehicle (Control) in complete culture medium supplemented or not with DAG for 3, 7 and 10 days. **A**, **B**: Cell proliferation was determined by a colorimetric assay using the Cell Proliferation Reagent WST-1. The results are presented as mean ± SEM from 4 independent experiments performed in duplicate; * p < 0.05 versus the corresponding control.

In order to determine non-toxic dasatinib concentrations that could be used to perform MSC differentiation assays, MSC were incubated with increasing concentrations of dasatinib (from 10^-9 ^to 10^-4 ^M) for 3, 7 or 10 days. Changes in cell proliferation were measured by WST-1 assay. Dasatinib induced a concentration-dependent decrease in cell proliferation: it had no effect at the lowest tested concentrations (10^-9 ^and 10^-8 ^M), had low inhibitory effects between 10^-7 ^and 10^-5 ^M and was clearly toxic at 10^-4 ^M (Fig. 4A), with morphological evidences of necrotic cell death (data not shown). Of note, DAG induced a weak inhibition of cell proliferation, but it did not change the concentration-related toxicity of dasatinib in MSC (Fig. 4B). Therefore, 10^-8 ^M dasatinib was identified as the highest non-toxic concentration to be used in differentiation assays.

### Effect of dasatinib on Src phosphorylation in MSC

**Figure 5:**
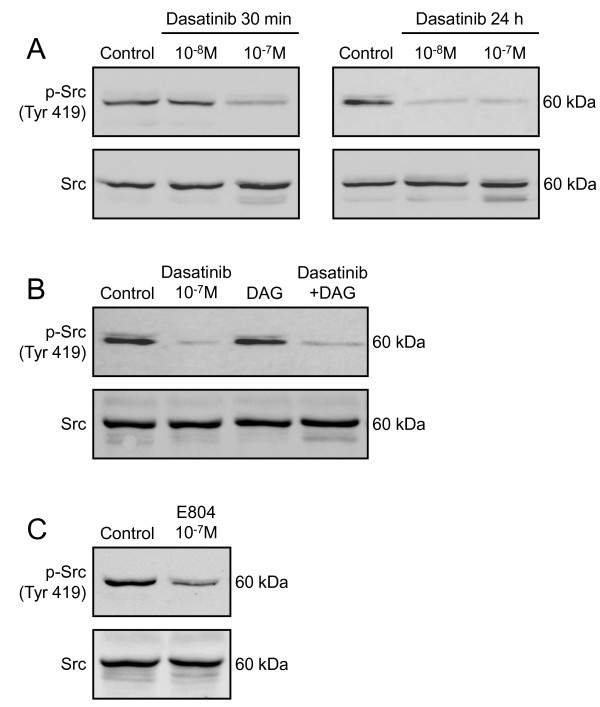
**Effect of dasatinib or E804 on Src phosphorylation**. **A**: Cells treated for 30 min or 24 hours with 10^-8^, 10^-7 ^M dasatinib or vehicle (Control) in complete culture medium. **B**: Cells exposed to 10^-7 ^M dasatinib and/or DAG or vehicle for 24 hours. **C**: Cells stimulated for 24 hours with 10^-7 ^M E804 or vehicle. **A**, **B**, **C**: Proteins (30 μg) were resolved by 10% SDS-PAGE and blotted onto nitrocellulose membranes. Immunodetections were performed using p-Src (Tyr 419) or Src antibodies, secondary antibodies and chemiluminescent substrate. Representative results from 2 independent experiments.

The activity of Src was evaluated by the level of Tyr 419 phosphorylation of Src in MSC exposed to 10^-8 ^and 10^-7 ^M dasatinib for 30 min or 24 hours. As shown in Fig. 5A, Src phosphorylation is significantly decrease in cells treated by 10^-7 ^M dasatinib for 30 min and in cells incubated with both drug concentrations for 24 hours, confirming that Src is a target of dasatinib in MSC. Importantly, DAG did not affect neither the Src phosphorylation nor the inhibition of Src phosphorylation induced by 10^-7 ^M dasatinib for 24 hours (Fig. 5B), indicating that dasatinib completes the effect of DAG on MSC differentiation by acting on Src. As positive control, the Src inhibitor E804 (10^-7 ^M, 24 hours) strongly decreased the phosphorylation of the tyrosine kinase (Fig. 5C).

### Effect of dasatinib on calcium deposition in MSC

**Figure 6:**
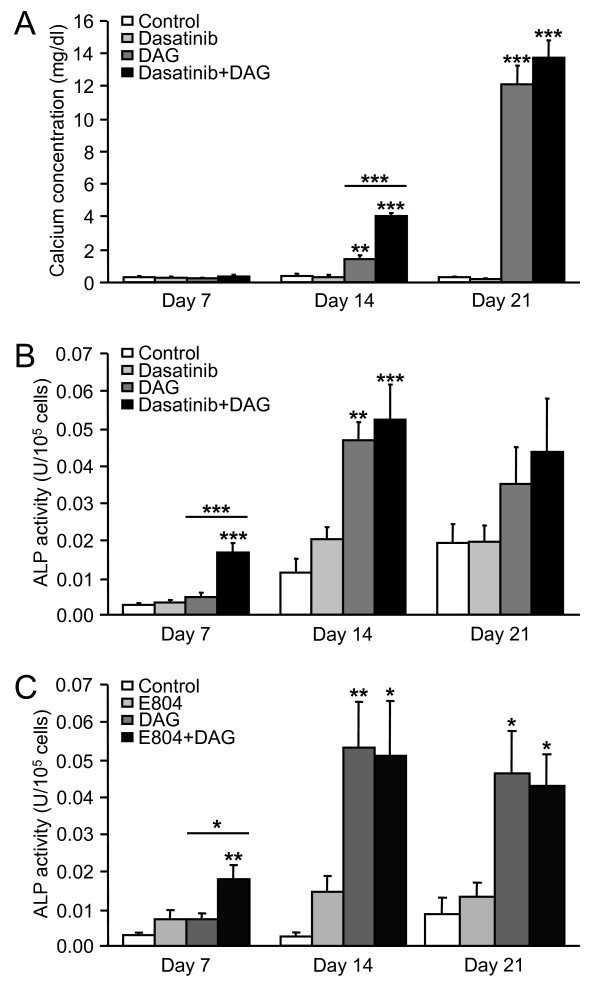
**Quantitative determination of matrix calcification and alkaline phosphatase activity in MSC exposed to dasatinib or E804**. **A**: Matrix calcium content of untreated MSC (Control), and cells cultured with 10^-8 ^M dasatinib, DAG or a combination of both treatments, was assessed after 7, 14 and 21 days. **B**: ALP activity in MSC cultured in control medium supplemented or not with 10^-8 ^M dasatinib and/or DAG was measured after 7, 14 and 21 days. ALP activity (U, μmol p-nitrophenol released per min) was normalized. **C**: ALP activity in MSC exposed to 10^-7 ^M E804 and/or DAG for 7, 14 and 21 days. ALP activity (U, μmol p-nitrophenol released per min) was normalized normalized for 10^5 ^cells. **A**, **B**, **C**: Results are presented as mean ± SEM from 4 independent experiments performed in duplicate; * p < 0.05, ** p < 0.01 and *** p < 0.001 versus the corresponding control, and comparing DAG versus Dasatinib+DAG or versus E804+DAG.

Mineralization of the extracellular matrix by MSC was determined by colorimetric assays after 7, 14 and 21 days of culture in control or osteogenic medium, supplemented or not with 10^-8 ^M dasatinib (Fig. 6A). Calcium deposition was very low after 7 days in all culture conditions. Calcification was observed after 14 days in cells cultured with DAG where dasatinib significantly increased the mineralization induced by DAG (apparent synergism). After 21 days, matrix calcification was further enhanced in presence of DAG, but dasatinib did not affect these high levels of calcium deposition. Thus, dasatinib alone was unable to affect matrix mineralization, but it may accelerate the process of calcification in presence of DAG.

### Effect of dasatinib on alkaline phosphatase activity in MSC

The activity of ALP was evaluated in MSC after 7, 14 and 21 days of culture with or without DAG and 10^-8 ^M dasatinib (Fig. 6B). After 7 days, dasatinib alone and DAG alone did not affect ALP activity, while the combination of both treatments significantly increased ALP activity (apparent synergism). At 14 and 21 days, ALP activity was higher than at 7 days in all culture conditions, and dasatinib failed to induce any additional stimulation of ALP activity over DAG. Hence, dasatinib may increase ALP activity early in the osteoblastic differentiation process of MSC. Importantly, the Src inhibitor E804 (10^-7 ^M) similarly potentiated the effect of DAG, increasing ALP activity after 7 days (Fig. 6C). These data strongly support that the effect of dasatinib in MSC was mediated through the inhibition of Src.

### Effect of dasatinib on osteoblast-related gene expression in MSC

**Figure 7:**
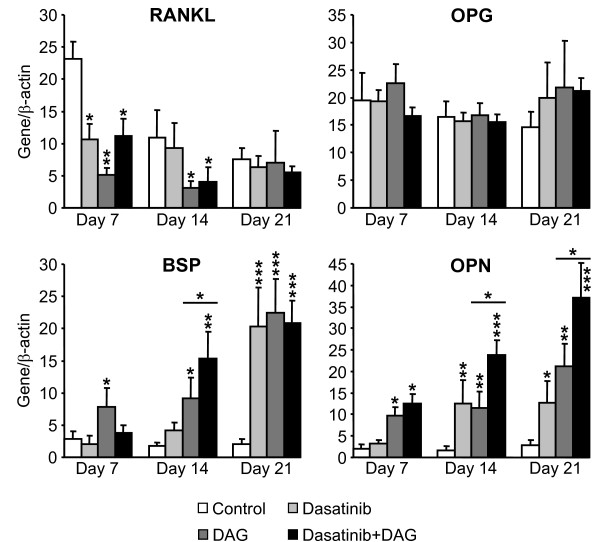
**Quantitative evaluation of expression of osteoblast-related genes in dasatinib-treated MSC**. Cells were treated or not with 10^-8 ^M dasatinib and DAG, alone or in combination. RNA was isolated from MSC and subjected to real-time quantitative PCR using specific primers for osteoblast gene markers (RANKL, OPG, BSP, OPN) and β-actin (see Table 2). Data are presented as mean ± SEM from 4 independent experiments performed in duplicate; * p < 0.05, ** p < 0.01 and *** p < 0.001 versus the corresponding control, and comparing DAG versus Dasatinib+DAG.

We identified 3 osteoblast-related genes (RANKL, BSP and OPN) whose levels of expression are informative of the osteoblastic differentiation status of MSC (see above). The expression of these genes reflecting MSC differentiation status was assessed by real-time quantitative PCR in MSC treated or not with DAG and exposed or not to 10^-8 ^M dasatinib (Fig. 7). Dasatinib and DAG, either alone or in combination, significantly decreased the expression of RANKL after 7 days of culture, but dasatinib did not induce additional effects on RANKL expression after 14 and 21 days. However, OPG mRNA expression did not significantly change during osteoblastic differentiation of MSC, indicating that the RANKL/OPG ratio variations paralleled the changes in RANKL. On the other hand, dasatinib alone significantly increased the expression of BSP after 21 days, while it enhanced OPN expression after 14 and 21 days. In addition, dasatinib also increased the DAG-induced upregulation of BSP after 14 days and of OPN after 14 and 21 days. Therefore, dasatinib was able to modulate the expression of osteoblast-related genes in a time-dependent manner, and it may also potentiate the effects of DAG on BSP and OPN expressions.

### Effect of dasatinib on RANKL/OPG ratio in MSC

**Figure 8:**
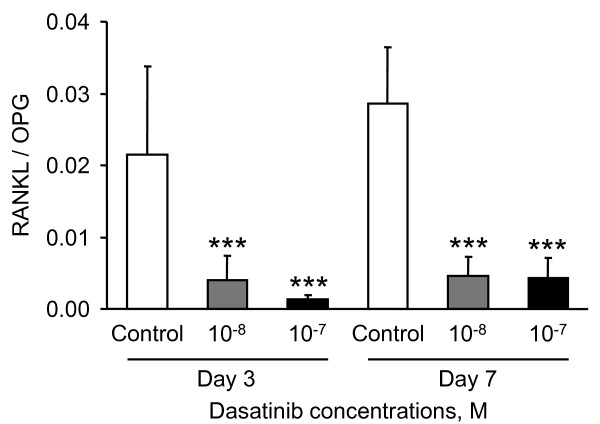
**Evaluation of RANKL/OPG ratio by ELISA in conditioned medium of MSC exposed to dasatinib**. Cells were treated or not with 10^-8 ^and 10^-7 ^M dasatinib in the absence of DAG for 3 and 7 days. 24-hours conditioned medium were collected at the end of the treatment time. RANKL and OPG protein levels were measured by ELISA in conditioned medium (1 ml/10^5 ^cells) and normalized to total protein content. Data are presented as RANKL/OPG ratio (mean ± SEM from 4 independent experiments). *** p < 0.001 versus the corresponding control.

To further investigate the effects of dasatinib on the expression of RANKL at protein level in short-term experiments, RANKL protein was evaluated by ELISA in 24-hours conditioned medium of MSC exposed to 10^-8 ^and 10^-7 ^M dasatinib, in absence of DAG, for 3 and 7 days. OPG protein was also measured by ELISA in the same samples and results are presented as RANKL/OPG ratio (Fig. 8). Data indicated that both concentrations of dasatinib significantly decreased the RANKL/OPG ratio at days 3 and 7, resulting from a weak and not significant increase in OPG expression (e.g. 1.7 fold, 10^-7 ^M drug, day 3) and a dramatic decrease in RANKL expression (e.g. 9 fold, 10^-7 ^M drug, day 3). This result strongly confirms our above observation at mRNA level.

## Discussion

We assessed the effects of the Src inhibitor dasatinib on the osteogenic differentiation of bone marrow-derived mesenchymal stromal cells (MSC) treated or not with a combination of dexamethasone, ascorbic acid and β-glycerophosphate (DAG).

Firstly, we have searched for the most informative markers of osteogenic differentiation of MSC induced by DAG and found a set of 5 markers (mineralization, ALP activity and expression of RANKL, BSP and OPN) that best characterize the 4 stages of osteoblast differentiation: native stage (undifferentiated MSC), early stage (osteoprogenitors), intermediate stage (mature osteoblasts), and late stage (late osteoblasts). Of note, many genes related to the osteoblastic phenotype or activity have been previously used to define the osteoblastic status of different cellular types [30,31]. However, in our model, we found that the expression of several of them, notably Runx2, COL-I, OSN and OPG, was already present in DAG-untreated MSC and remained unchanged during osteoblastic differentiation, while others have reported that the expression of Runx2 and COL-I increased along the osteoblast differentiation [32]. This divergence can be explained by the fact that MSC preparations may contain multidifferentiated cells, which express both immature and mature proteins of different tissues [33,34]. In addition, recent reports indicate that the phosphorylation level but not the expression level of Runx2 is related to the differentiation status of osteoblasts [35]. Therefore, the expression of these genes should be considered rather as a weak indicator of MSC differentiation into osteoblasts. Interestingly and in accordance with previous observations [36,37], ALP activity and gene expression increased during MSC differentiation into osteoblasts. However, we found that ALP activity and gene expression did not fully correlate; ALP activity is probably more reliable to the mineralization process. Indeed, in undifferentiated MSC and late osteoblasts, ALP activity was, respectively, very low and significantly downregulated, while ALP mRNA was expressed at high levels. These data indicate that ALP activity is more informative than ALP gene expression as a marker of MSC differentiation.

Based on our selection of markers, we have evaluated the effects of the Src inhibitor dasatinib on the differentiation of MSC into osteoblasts. Our interest in Src inhibitors stems from a recent study showing that the differentiation of the MC3T3-E1 pre-osteoblasts was accompanied by a decrease in phosphorylation of the activator site (^416^Tyr) of Src and an increase in phosphorylation of its inhibitor site (^527^Tyr) [38]. This indicates that the post-translational inhibition of Src is associated with the activation of osteoblast differentiation. Moreover, targeted disruption of Src gene in mice leads to osteopetrosis [39], and osteoblasts isolated from Src^-/- ^mice exhibit accelerated differentiation and elevated levels of osteoblast-related markers [40]. Taken together, these data suggest that Src inhibition may stimulate osteoblast differentiation. To validate that Src was targeted by dasatinib in MSC, we confirmed that dasatinib inhibits the phosphorylation of the activator site of Src. We also showed that the specific Src inhibitor E804 stimulated osteoblast differentiation as it increased ALP activity in MSC exposed to DAG. By contrast, mice deficient in Abl, another potent target of dasatinib, are osteoporotic and have defects in osteoblast maturation [41], excluding a positive role of Abl inhibition by dasatinib in the differentiation of MSC into osteoblasts. We also considered other targets of dasatinib, namely platelet-derived growth factor receptor (PDGFR) [42] and cKIT/CD117 [43]. The role of PDGFR in the osteoblastic differentiation of MSC remains, however, controversial [44,45], and new data clearly demonstrated that PDGFR signaling is not involved in osteogenic differentiation of human MSC, while it affects MSC proliferation [46]. On the other hand, we did not detect cKIT in MSC (data not shown, Western blotting using mouse mAb cKIT (Ab81) from Cell Signaling Technology, LND1 melanoma cell line as positive control), confirming previous observations [47]. Altogether, these data indicate that Src is the most relevant dasatinib target involved in the process of osteoblastic differentiation of MSC.

In this study, we found that dasatinib promoted time-dependent changes in osteoblast-related markers in MSC. These changes matched the ones induced by DAG and corresponded to the progressive differentiation of MSC into osteoblasts (see Fig. 3). Indeed, we documented that dasatinib, in combination with DAG, increased ALP activity after 7 days, and consequently, speeded up calcium deposition after 14 days without exceeding the maximum levels reached with DAG alone, and that it increased the expression of BSP and OPN, mainly in the late stage of MSC differentiation. Thus, dasatinib was able to stimulate each stage of the differentiation of MSC into osteoblasts. Importantly, our experiments were performed with a non-toxic but effective concentration of dasatinib (10^-8 ^M) able to inhibit Src kinase activity (inhibition of Src phosphorylation) in accordance with a previous report showing inhibition of the kinase activity of purified Src protein with an IC50 of 3 × 10^-9 ^M [27]. Altogether, our data, added to previous studies from other groups [20,22,23,27], strongly support that Src kinase activity is the main target for dasatinib in MSC differentiation process. Interestingly, a very recent study revealed that dasatinib strongly enhanced differentiation of primary mouse osteoblasts isolated from mouse calvaria (stimulation of ALP activity, osteocalcin secretion, matrix mineralization) [29]. The authors showed that among the Src family kinases, only Src was activated at a high level in this model, and that ^419^Tyr-Src phosphorylation was inhibited by dasatinib. Moreover, knockdown of Src by lenti-shRNA in osteoblasts enhanced their differentiation, suggesting that dasatinib stimulated osteoblast differentiation through the inhibition of Src. Of note, the authors also reported that osteoblast differentiation by dasatinib could be mediated through the inhibition of Abl, however, they found that Abl was expressed at a low level in osteoblasts, suggesting a limited impact.

In the other hand, we observed that dasatinib alone was able to decrease RANKL mRNA expression as well as RANKL protein released in culture medium (decrease in RANKL/OPG ratio) in undifferentiated MSC (no DAG induction) cultured up to 7 days, suggesting a possible indirect inhibitory effect on osteoclastogenesis and, consequently, on bone resorption. In this context, recent studies reported that tyrosine kinase inhibitors are effective in inhibiting the differentiation and activity of human osteoclasts [48], and in preventing bone loss in animal models [17]. Thus, our data indicate that, in addition to its previously reported direct inhibitory effects on the osteoclast formation and activity [49,50], dasatinib may also act indirectly on osteoclasts through the modulation of RANKL expression in osteoblasts.

Furthermore, as far as the bone loss associated with metastases is concerned, recent preclinical and clinical studies indicated that dasatinib could potentially have inhibitory effects both on osteoclasts and tumor cells. Indeed, dasatinib decreased the osteolysis induced by prostate cancer cells injected into tibiae of SCID mice and inhibited the growth of cancer cells [51], while it reduced the expression of markers of bone resorption in patients with advanced castration-resistant prostate cancer [52]. Thus, our findings complete these observations by demonstrating a direct effect of dasatinib on osteoblasts, which could further contribute to the disruption of the vicious circle established between bone cells and tumor cells.

Our data also suggest that patients with bone loss could benefit from dasatinib therapy in accordance with critical findings using imatinib, another tyrosine kinase inhibitor currently used in chronic myeloid leukemia (CML). Indeed, short-term imatinib treatment increased OPG/RANKL ratio and osteocalcin levels in serum of CML patients [53], while long-term (> 2 years) imatinib therapy promoted bone formation [54] and increased bone mineral density (cortical bone mineralization) [55].

## Conclusions

Our study reports an original dual effect of dasatinib on MSC at non toxic concentrations: (i) it is able to significantly speed up the osteogenic differentiation of MSC, and (ii) it can inhibit RANKL expression in undifferentiated MSC (no DAG induction) thus preventing osteoclast activation. This dual effect on osteoblasts, on top of its direct activity on osteoclasts as well as on tumor cells, may provide a therapeutic benefit in patients with diseases leading to bone loss, such as osteoporosis, tumor-induced osteolysis, and therapy-induced bone loss.

## Competing interests

MP and JJB are recipients of a research grant from Bristol Myers Squibb (Princeton, NJ, USA).

## Authors' contributions

HIB participated in experimental design, performed experiments and analyses. LL participated in experimental design, interpreted the data, and drafted the manuscript. MN helped to draft the manuscript. MP, GG and JJB discussed the results and critically revised the manuscript. FJ designed the experiments, assembled tables and figures, and revised the manuscript. All authors read and approved the final manuscript.

## Pre-publication history

The pre-publication history for this paper can be accessed here:

http://www.biomedcentral.com/1471-2407/10/298/prepub
